# Transcriptional Profiling of Hilar Nodes from Pigs after Experimental Infection with Actinobacillus Pleuropneumoniae

**DOI:** 10.3390/ijms141223516

**Published:** 2013-11-29

**Authors:** Shumin Yu, Zhicai Zuo, Hengmin Cui, Mingzhou Li, Xi Peng, Ling Zhu, Ming Zhang, Xuewei Li, Zhiwen Xu, Meng Gan, Junliang Deng, Jing Fang, Jideng Ma, Shengqun Su, Ya Wang, Liuhong Shen, Xiaoping Ma, Zhihua Ren, Bangyuan Wu, Yanchun Hu

**Affiliations:** 1 College of Veterinary Medicine, Sichuan Agricultural University, Ya’an 625014, China; E-Mails: yayushumin@163.com (S.Y.); zzcjl@126.com (Z.Z.); pengxi197313@163.com (X.P.); abtczl72@126.com (L.Z.); abtcxzw@126.com (Z.X.); gm0320@163.com (M.G.); dengjl213@126.com (J.D.); fangjing4109@163.com (J.F.); wangyayang@126.com (Y.W.); shenliuhong@sohu.com (L.S.); mxp886@sina.com (X.M.); zhihua_ren@126.com (Z.R.); wubangyuan2008@163.com (B.W.); hychun114@163.com (Y.H.); 2 Laboratory of Animal Disease and Human Health, Sichuan Agricultural University, Ya’an 625014, China; 3 College of Animal Science and Technology, Sichuan Agricultural University, Ya’an 625014, China; E-Mails: mingzhou.li@163.com (M.L.); zhm3000@163.com (M.Z.); xuewei.li@sicau.edu.cn (X.L.); jideng_ma@sina.com (J.M.); 4 Library of Sichuan Agricultural University, Ya’an 625014, China; E-Mail: zzcjl@tom.com

**Keywords:** porcine pleuropneumonia, infection, *Actinobacillus pleuropneumoniae*, Agilent Porcine Genechip, microarray analyses, cytokine, host defense response

## Abstract

The gram-negative bacterium *Actinobacillus pleuropneumoniae* (*APP*) is an inhabitant of the porcine upper respiratory tract and the causative agent of porcine pleuropneumonia (PP). In recent years, knowledge about the proinflammatory cytokine and chemokine gene expression that occurs in lung and lymph node of the *APP*-infected swine has been advanced. However, systematic gene expression profiles on hilar nodes from pigs after infection with *Actinobacillus pleuropneumoniae* have not yet been reported. The transcriptional responses were studied in hilar nodes (HN) from swine experimentally infected with *APP* and the control groupusing Agilent Porcine Genechip, including 43,603 probe sets. 9,517 transcripts were identified as differentially expressed (DE) at the *p* ≤ 0.01 level by comparing the log2 (normalized signal) of the two groups named treatment group (TG) and controls (CG). Eight hundred and fifteen of these DE transcripts were annotated as pig genes in the GenBank database (DB). Two hundred and seventy-two biological process categories (BP), 75 cellular components and 171 molecular functions were substantially altered in the TG compared to CG. Many BP were involved in host immune responses (*i.e.*, signaling, signal transmission, signal transduction, response to stimulus, oxidation reduction, response to stress, immune system process, signaling pathway, immune response, cell surface receptor linked signaling pathway). Seven DE gene pathways (VEGF signaling pathway, Long-term potentiation, Ribosome, Asthma, Allograft rejection, Type I diabetes mellitus and Cardiac muscle contraction) and statistically significant associations with host responses were affected. Many cytokines (including *NRAS*, *PI3K*, *MAPK14*, *CaM*, *HSP27*, *protein phosphatase 3*, *catalytic subunit and alpha isoform*), mediating the proliferation and migration of endothelial cells and promoting survival and vascular permeability, were activated in TG, whilst many immunomodulatory cytokines were suppressed. The significant changes in the expression patterns of the genes, GO terms, and pathways, led to a decrease of antigenic peptides with antigen presenting cells presented to T lymphocytes via the major histocompatibility complex, and alleviated immune response induced *APP* of HN. The immune response ability of HN in the *APP*-infected pigs was weakened; however, cell proliferation and migration ability was enhanced.

## 1. Introduction

Members of the *Actinobacillus genus* (family Pasteurellaceae) are Gram-negative facultative anaerobic bacteria. They range from commensal species of the mammalian respiratory and genital tracts to important pathogens [1,2]. *Actinobacillus pleuropneumoniae* (*APP*) is the causative agent of porcine pleuropneumonia (PP), a disease occurring worldwide and causing significant economic losses in the swine industry [3–7]. The disease, which occurs in swine of all ages, is highly infectious, often fatal, and characterized by necrotizing, hemorrhagic bronchopneumonia and serofibrinous pleuritis [8]. *APP* can spread quickly by air-borne particles and/or touching contaminated surface, and often kill infected animals in the acute phase when extensive lung hemorrhage and necrosis occur. Swine that survived often developed pleurisy, the sequelaes of local necrosis of the pleura, or became healthy carriers of *APP. APP* can be divided into two biovars based on its nicotinamide adenine dinucleotide (NAD) requirements: biovar 1, which is NAD dependent, and biovar 2, which can synthesize NAD in the presence of specific pyridine nucleotides or their precursors [4].

The porcine lung infected with APP has previously been reported to result in local production of proinflammatory proteins or to mRNA encoding the cytokines *interleukin (IL)-1α*, *IL-1β*, *IL-6* and the chemokine *IL-8* [9,10]. Likewise, bioactive protein and/or mRNA coding for *IL-10*, *IL12p35*, *TNF-α* and *IFN-α* had been shown to be up-regulated after infection with APP *in vivo* or *in vitro* [9–15]. Using cDNA microarrays, Moser and co-workers identified 307 anonymous transcripts in blood leukocytes obtained from pigs that were severely affected by experimental infection with APP [16]. Hedegaard *et al*. investigated the molecular characterization of the early response in pigs to infection with APP serotype 5B, using cDNA microarrays [17]. In this study, Hedegaard *et al*. found three subsets of genes that were consistently expressed at different levels depending upon the infection status and a total of 130 genes had different expression profiles in hilar node (HN) tissue samples from infected versus non-infected animals [17]. Mortensen *et al*. studied the local transcriptional response in different locations of lung from pigs experimentally infected with the respiratory pathogen APP 5B, using porcine cDNA microarrays (DJF Pig 55 K v1) representing approximately 20,000 porcine genes printed in duplicate [18]. Within the lung, Mortensen *et al*. found a clear division of induced genes as, in unaffected areas a large part of differently expressed genes were involved in systemic reaction to infection, while differently expressed genes in necrotic areas were mainly concerned with homeostasis regulation [18]. Zuo *et al*. studied the relationship between infection and injury by investigating the whole porcine genome expression profiles of swine lung tissues post-inoculated with experimental *APP*, and found 11,929 transcripts differentially expressed at the *p* ≤ 0.01 level [19]. Many proinflammatory-inflammatory cytokines were activated and involved in the regulation of the host defense response at the site of inflammation; while the cytokines involved in regulation of the host immune response were suppressed [19].

*APP* often kill infected swine in the acute phase, the lung and pleural of dead animals character by necrotizing, hemorrhagic bronchopneumonia and serofibrinous pleuritis. HN is one of the important immune organs adjacent to lung. Thus, transcriptional profiling of whole porcine genome in HN sampled from inoculated *versus* non-inoculated swine would lead to greater knowledge of the host response dynamics to bacterial infection in the lung. This knowledge is important to obtain a more complete picture of the lung-specific host reaction in the pathogenesis of respiratory infection.

In the present study, the Agilent Whole Porcine Genome Oligo Microarrays were used to detect the changes in gene expression of infected pigs’ HN from non-inoculated animals. Ten transcripts (the top six up-regulated and the top four down-regulated in microarray data) were selected to verify the accuracy and reproducibility of the microarray data by real-time quantitative reverse transcription-polymerase chain reaction (qRT-PCR).

## 2. Results

### 2.1. Clinical Symptoms and Necropsy Findings

Swines showed hyperthermia (40.6–42.0 °C), dyspnea and anorexia after being inoculated with *APP* 24–48 h. Two swines died with respiratory distress at post-inoculation 36–48 h. In the autopsy, the lungs were severely damaged by acute, multifocal, fibrino-necrotizing and hemorrhagic pneumonia complicated by acute diffuse fibrinous pleuritis. The HN were enlarged and congested. No lesions in CG were observed. The infected swines had lung and pleural lesions of variable severity consistent with acute pleuropneumonia, whereas the surrounding lung and pleural tissue appeared normal.

**Figure 1.**
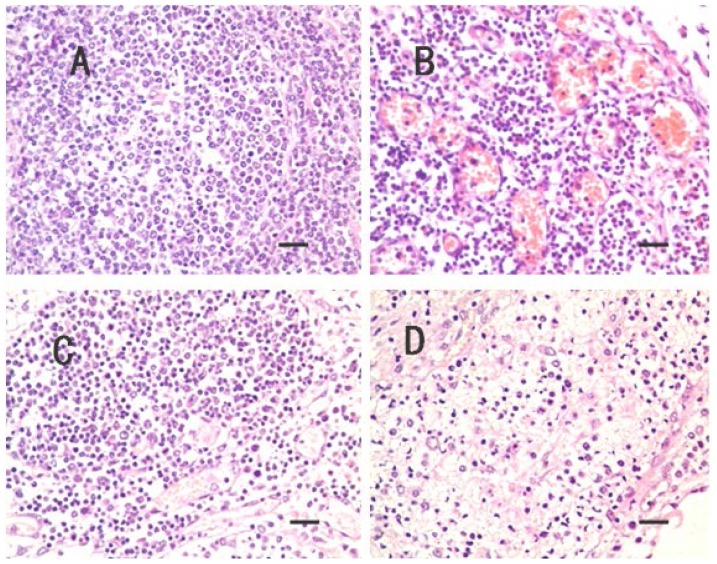
No lesions were observed in control group (CG) hilar nodes (HN) (**A**) (scale bar = 25 μm, 400×); The HN in treatment group (TG), the little veins are congested in medulla (**B**) (scale bar = 25 μm, 400×); and the little veins and capillaries are congested in cortex and medulla (**C**) (scale bar = 25 μm, 400×); and the medulla are obvious edema, and neutrophils infiltration (**D**) (scale bar = 25 μm, 400×).

Histopathological lesions were not observed in CG. However, lesions in TG’s in TG’s HN were characterized by loose medulla, congestion, edema, fibrinous exudation and neutrophils infiltration (Figure 1).

### 2.2. Microarray Profiling

Expression profiling was conducted using a commercially available Agilent Porcine Genechip that included 43,603 probe sets. The transcriptome of the HN was determined. Expression was detected for 29,105 probes (66.75% of all probe sets) of the CG. A total of 29,646 probes (67.99% of all probe sets) were expressed in TG. When probe sets’ intensities had been normalized and filtered, there were still 26,353 probes used to identify significantly DE genes. There were 9,517genes identified as DE at the *p* ≤ 0.01 level by comparing the log2 (normalized signal) of the two groups (CG *vs*. TG) using *t*-test analysis.

**Figure 2.**
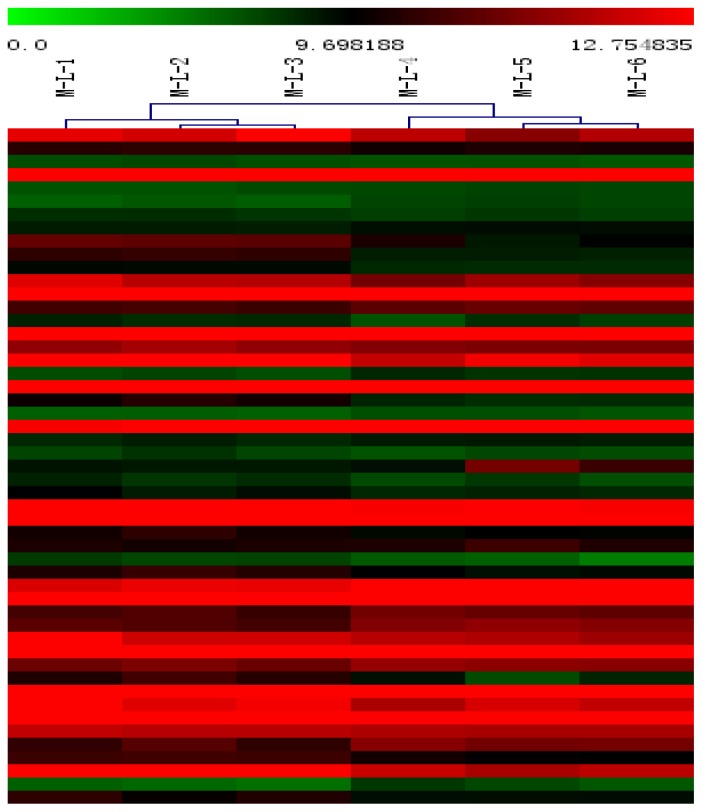
Hierarchical clustering analysis and clustering segmentation.

Hierarchical clustering was applied to the mean log-ratio of the replicated spots from the DE genes by the average linkage and used euclidean distance as the similarity metric (Figure 2). The expression profiles of samples were divided into two groups- one from the non-inoculated swines (M-L-1, M-L-2, M-L-3) and the other group from the inoculated swine (M-L-4, M-L-5, M-L-6).

**Figure 3.**
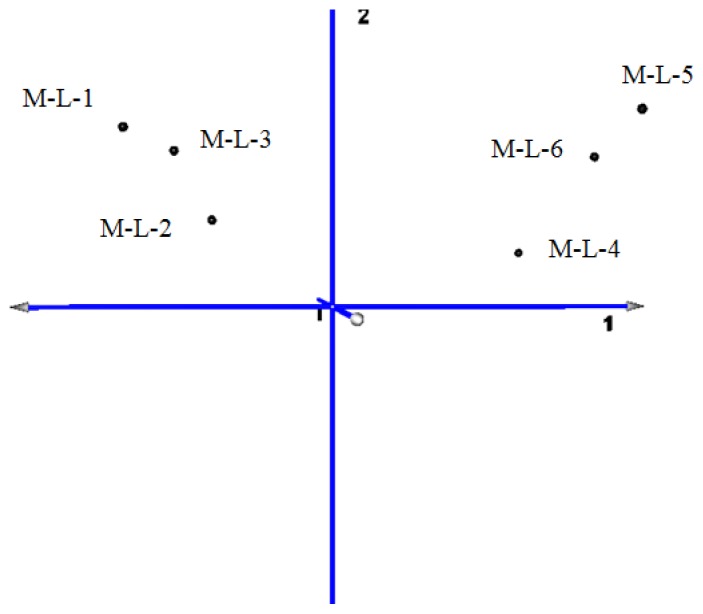
Three-dimensional map of principal component analysis (PCA) for mapping samples obtained from clustering segmentation.

The principal component map to three-dimensional space, also found that the distance of CG (samples M-L-1, M-L-2, M-L-3) is short, and the gene expression pattern is more consistent. The distance of TG (samples M-L-4, M-L-5, M-L-6) is relatively discrete because of the differences in the lesions degree. The gene expression pattern is similar (Figure 3).

The six samples were set as variables. The principal component analysis (PCA) of the co-expressed differentially genes (CG *vs*. TG) showed that the contribution rate of the first principal component of which has reached 97.16%. The total contribution rate of the first three principal components reached 99.41% (Table 1).

### 2.3. Differentially Expressed (DE) Gene Analysis

Of the 9,517 DE genes, 815 were annotated as pig genes in the GenBank database (DB). GO and KEGG pathway analyses of the 815 DE gene lists were conducted using DAVID. There were 272 biological process (BP) categories (Supplementary Table S1), 75 cellular components (Supplementary Table S2), and 171 molecular functions (Supplementary Table S3) were significantly affected by infection with *APP* (*p* = 0). There were many BP related to the immune responses, and a number of BP related to metabolism were also identified.

A total of 344 genes were analyzed by pathway enrichment analysis (GSEA). Two hundred and thirty-eight genes (69.19% of the total) were correlated with TG, while the other 106 (30.81%) genes were correlated with CG. One hundred and forty-two of the total 170 pathways remained for further analysis after size filtering (2 ≤ sizes ≤ 20). Altogether, 104/142 pathways (Supplementary Table S4) were enriched and up-regulated in the TG and down-regulated in the CG. Two pathways (namely, VEGF signaling pathway and Long-term potentiation) were significantly enriched at nominal *p* values of less than 1% and 5%.

Thirty-eight pathways (Supplementary Table S5) were down-regulated in the TG. One pathway (Allograft rejection) was statistically significant at a false discovery rate of <25%. Five pathways (namely, Ribosome, Asthma, Allograft rejection, Type I diabetes mellitus and Cardiac muscle contraction) were significantly enriched at a nominal *p* value of less than 1% and 5%.

**Figure 4.**
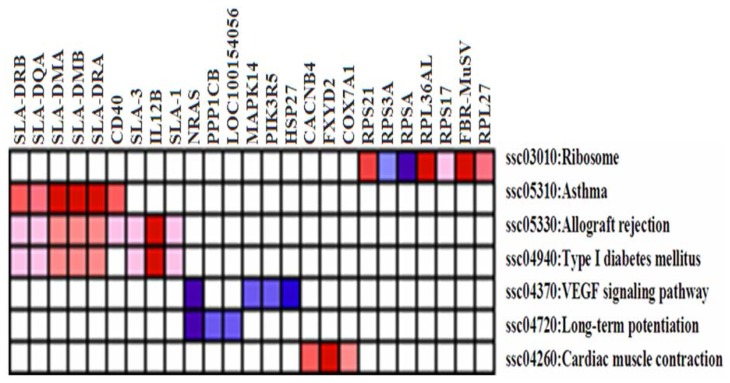
The heat map shows the clustered genes in the leading edge subsets. In the heat map, expression values are represented as colors, where the range of colors (red, pink, light blue, dark blue) represents the range of expression values (high, moderate, low, lowest) in the CG. This pattern is reversed in the TG.

Further analysis revealed that several genes were induced by leading edge analysis for the seven significant pathways (Figure 4). The genes included those encoding *v-Ha-ras Harvey rat sarcoma viral oncogene homolog (NRAS)*, *protein phosphatase 1 (PP1)*, *similar to calmodulin (CaM)*, *similar to mitogen-activated protein kinase 14 (MPK14)*, *p101 protein (PI3K)*, *heat shock 27 kDa protein 1 (HSP27)*, *ribosomal protein (RP) S3A* and *RP SA*, among others. The repressed genes comprised those encoding members of the *major histocompatibility complex (MHC)*, including *(SLA-1*, *SLA-3*, *SLA-DRA*, *SLA-DRB1*, *SLA-DMB*, *SLA-DQA*, *SLA-DMA)*, *CD40*, *IL-12B*, *calcium channel*, *voltage-dependent*, *beta 4 subunit (CACNB4)*, *FXYD domain containing ion transport regulator 2 (FXYD2)*, *cytochrome c oxidase polypeptide VIIa-muscle/heart (COX7A1)*, *Finkel-Biskis-Reilly murine sarcoma virus (FBR-MuSV) ubiquitously expressed (Fau)*, *RP L27*, *RP21*, *RP L36a-like*, *RP S17*, among others.

Genes that were frequently induced in the TG included NRAS, while genes such as *SLA-1*, *SLA-3*, *SLA-DRA*, *SLA-DRB*, *SLA-DMB*, *SLA-DQA*, *SLA-DMA*, *CD_40_*, *IL-12B* were frequently suppressed.

### 2.4. Verification of Gene Expression Pattern from Microarray Data Using Real-Time QRT-PCR

**Figure 5.**
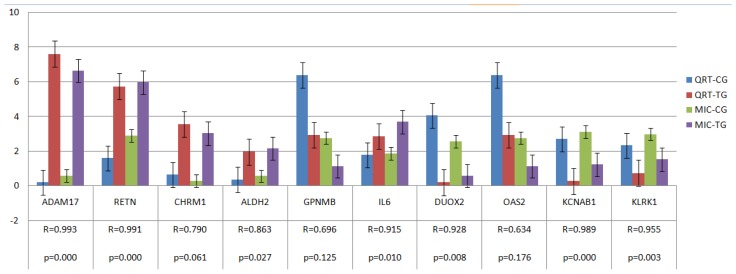
Validation of the microarray data by the real-time qRT-PCR analyses of ten representative genes. The *x*-axis represents the genes and the y-axis shows their relative expression levels (−Δ*C*_t_) values for quantitative real-time RT-PCR; Log (Sample signal, 10) for microarray). Three biological replicates were conducted for both assays. R represents the Pearson correlation coefficient. The significance of differences for gene expression between the CG and the TG was calculated using a two-tailed *t*-test. MIC-TG or CG on the right side of the figure represents microarray data in TG or CG, and QRT-TG or CG represents Quantitative real-time polymerase chain reaction data in TG or CG.

Ten genes (namely, *RETN*, *ADAM17*, *GPNMB*, *CHRM1*, *ALDH2*, *IL6*, *KLRK1*, *DUOX2*, *OAS2* and *KCNAB1*) were selected to confirm expression patterns using real-time qRT-PCR. The results indicate that the expression patterns of all the genes were consistent with the microarray data (*r* = 0.875 ± 0.122, Figure 5).

## 3. Discussion

In the present study, we revealed 9,517 DE genes in HN using Agilent Whole Porcine Genome Oligo Microarrays (one-color platform) that contain 43,603 probes, while 11,929 DE genes in lung tissue [19]. There were 815 genes annotated as swine genes in the GenBank Data Base (DB). GO term analysis identified that a total of 272 BP categories, 75 cellular components and 171 molecular functions were significantly affected and at least 50 BP categories in HN were related to the host immune response (*i.e.*, signaling, signal transmission, signal transduction, response to stimulus, oxidation reduction, response to stress, immune system process, signaling pathway, immune response, cell surface receptor linked signaling pathway), while a total of 89 BP categories, 82 cellular components and 182 molecular functions were significantly affected and 27 BP categories were related to the host immune response in lung [19]. Thus, the body in the course of anti-infective, immune organs adjacent to the infected tissue played more complex biological role, especially immunomodulatory effects.

The environmental information processing and signal transduction pathways (including Long-term potentiation, VEGF signaling pathway, the erbB signaling pathway, the cnt signaling pathway, and the calcium signaling pathway), host immune responses pathways (including Toll-like receptors signaling pathway, NOD-like receptors signaling pathway, RIG-I-like receptors signaling pathway, B cell receptor signaling pathway), cellular processes pathways (including the cell cycle, p53 signaling and apoptosis), and other pathways associated with metabolism (including the citrate cycle, Glycolysis/Gluconeogenesis, fatty acid metabolism, valine, leucine and isoleucine degradation andbeta-Alanine metabolism), *etc.*, were significantly enriched in inflamed HN. While the NOD-like receptors signaling pathway, Fc epsilon RI signaling pathway, acute myeloid leukemia, melanoma, progesterone-mediated oocyte maturation, spliceosome, type II diabetes mellitus and steroid hormone biosynthesis, *etc*., were significantly enriched in inflamed lung [19]. Five pathways, such as type I diabetes mellitus, autoimmune thyroid disease, allograft rejection, tight junction and cardiac muscle contraction were significantly enriched in non-inflamed lung [19]. The pathways activated in infected lung tissues were also including the acute myeloid leukemia pathway, progesterone-mediated oocyte maturation pathway, steroid hormone biosynthesis pathway, type II diabetes mellitus, spliceosome pathway and melanoma pathway [19].

Long-term potentiation and VEGF signaling pathway were two important pathways implicated in environmental information processing and signal transduction. Long-term potentiation can activate the Erk/MAP kinase and cAMP regulatory pathways [20]. VEGF signaling pathway can lead to a cascade of different signaling pathways, resulting in the up-regulation of genes involved in mediating the proliferation, migration of endothelial cells, promoting their survival and vascular permeability [21–24]. Specific families of pattern recognition receptors responsible for detecting microbial pathogens and generating innate immune responses, including *Toll-like receptors (TLRs)* [25–27], *NOD-like receptors (NLRs)* [28,29] and *RIG-I-like receptors (RLRs)* [30,31], were implicated in the activation of host immune responses pathways. Pathways repressed in inflamed HN, mainly including the MAPK signaling pathway, the TGF-β signaling pathway, antigen processing and presentation, focal adhesion and the adherents junction, amongst other.

Genes shown by leading edge analysis were activated in inflamed HN, including *NRAS* [32,33], *PI3K* [34–39], *MAPK14* [40], *CaM*, *HSP27*, *protein phosphatase 3 (formerly 2B)*, *catalytic subunit and alpha isoform (CALN)*, amongst others. Activations of all these genes were implicated in the proliferation and migration of endothelial cells promoting their survival and vascular permeability. *CaM* can lead to intracellular activation of N*RAS* and *CALN*, whilst *MAPK* promotes subsequent initiation of DNA synthesis and cell growth. In addition, *PI3K* leads to increasing endothelial-cell survival. Activation of *PI3K*, *HSP27* and *MAPK14* were implicated in cell migration signaling induced response cells as granulocytes (e.g., neutrophils, eosinophils, and basophils) and monocytes migration to the site of inflammation. While the gene encoding *IL-6*, *IL-8*, *IL-18*, *TNF*, *GM-CSF*, *CCL2*, *p101 protein*, *HLA-B associated transcript 1*, *Fc fragment of IgE*, *MAPK14*, *MAP2K1*, *IGF-1*, *STAT3* and *STAT5B*, *etc*., were activated at the site of inflammation lung [19]. Activations of all these genes can stimulate stem cells to produce granulocytes (neutrophils, eosinophils, and basophils) and monocytes, and also induce neutrophils and macrophages to phagocytose bacterial and foreign antigens [19].

Those genes encoding members of the *major histocompatibility complex (MHC)*, *CD40*, *IL-12B* were repressed in inflamed HN, while immunomodulatory cytokines *IL2*, *IL12B*, *CD40*, members of the *MHC* (*SLA-2*, *SLA-3*, *SLA-6*, *SLA-8*, *SLA-DRB1*, *SLA-DMB*, *SLA-DQA*, *SLA-DMA* and *SLA-DQB1*) were significantly suppressed at the site of inflamed lung [19]. *MHC* is a set of molecules (namely, *SLA-1*, *SLA-3*, *SLA-DRA*, *SLA-DRB1*, *SLA-DMB*, *SLA-DQA*, *SLA-DMA*) displayed on cell surfaces that can present antigenic peptides to T lymphocytes, which are responsible for a specific immune response that can destroy the pathogen producing those antigens [41]. Interactions between the CD_4_ molecule (found on helper T-cells), class II MHC or the CD_8_ molecule (found on cytotoxic T-cells) and class I MHC, act to stabilize and consummate the antigen recognition process. This allows helper T-cells to respond to “exogenous” antigens (leading to B-cell activation and the production of antibody), or cytotoxic T-cells to respond to “endogenous” antigens (leading to target cell destruction). *CD_40_* is a co-stimulatory protein found on antigen presenting cells (APCs) and is essential for mediating a broad variety of immune and inflammatory responses including T cell-dependent immunoglobulin class switching and memory B cell development [42–44]. *IL12B*, the gene encoding the p40 subunit of *IL-12* and *IL-23* [45], is a potent IFNγ-inducing cytokine secreted by macrophages and dendritic cells [46,47]. *IL-12/IL-23* signaling can influence host responses independent of *IFN-γ* and autocrine *IL-12/IL-23* production by APC [45]. *IL-12* is also an essential inducer of Th1 cell development, and has an important role sustaining a sufficient number of memory/effector Th1 cells to mediate Long-term protection against an intracellular pathogen [48]. Hence, down-regulation of Cytokines (*MHC*, *CD40* and *IL12B*) finally leads to a decrease in antigenic peptides presented to T lymphocytes by APCs via the MHC; this influences T cell function, memory B cell and Th1 cell development, and suppresses immune response.

Genes encoding metabolism and ribosomal proteins were affected, induced including *PP1* and *RP* (*S3A*, *SA*), repressed including *CACNB4*, *Fau*, *COX7A1* and *RP* (*S17*, *S21*, *L27*, *L36a-like*). Previous studies have shown that 41 out of 54 genes encoding *RP* were down-regulated in *Pseudomonas aeruginosa* after treatment with H_2_O_2_, a molecule which induces oxidative stress [49].

In the future studies, although additional work including more animals and time points is clearly needed to further delineate the host response to *APP* infection, the results obtained here demonstrate that: (1) a total of 272 biological process categories, 75 cellular components and 171 molecular functions were significantly affected by *APP*, and more than 50 biological process were involved in the host immune response against *APP*; (2) seven differentially expressed gene pathways had statistically significant associations with host responses in TG; (3) many of the cytokines mediating the proliferation and migration of endothelial cells and promoting their survival and vascular permeability were activated in TG; (4) several immunomodulatory cytokines were suppressed in TG. All changes of the genes, GO terms, and pathways which induced or repressed expression, led to decrease in antigenic peptides presented to T lymphocytes by antigen presenting cells (APC) via *MHC* and alleviated immune response injury induced by infection in HN. The immune response of HN in the *APP*-infected pigs was weakened; in contrast, cell proliferation and migration signaling was enhanced. Additional work including more animals and time points is clearly needed to further delineate the host response to APP infection and will contribute to a more detailed description of the dynamics of host responses in general.

## 4. Experimental Section

### 4.1. Animals, Bacterial Inoculation and Samples

All animal were conducted according to the Regulations for the Administration of Affairs Concerning Experimental Animals (Ministry of Science and Technology, China, revised in June 2004) and approved by the Institutional Animal Care and Use Committee in College of Animal Veterinary Medicine, Sichuan Agricultural University, Sichuan, Chinaunder permit No. DYY11966Twenty 12-week-old male castrated Danish Landrace/Yorkshire/Duroc crossbred swine from a healthy herd free from APP were divided equally into a control group (CG) and the treatment group (TG). APP serotype I (Strain provided by the Animal Biotechnology Center, Laboratory of Animal Disease and Human Health, Sichuan Agricultural University) was cultivated overnight at 37 °C in air on trypticase soy broth (TSB) (Hangwei, Hangzhou City, China). Bacterial counts of the suspensions were performed at the same time as the start of the inoculation. The inoculation was performed by holding the pigs (1–10) from the TG in an upright sitting position and spraying 0.25 mL diluent containing (3.5 – 4) × 10^7^ CFU/mL APP per kilogram weight into the nostrils during inspiration. Swine from the CG (swines 11–20) were inoculated with physiological saline (0.9% *w*/*v* NaCl) by the same means. In the TG, HN was collected 48 h post-inoculationfrom three swine (swines 1–3) after abattage and used for total RNA extraction and pathological analysis. Another three swine (swines 11–13) from the CG were sacrificed 48 h post-inoculation and their HN were collected by the same methods. The remaining swine were used for other trials.

### 4.2. Microarray Hybridizations and Data Analysis

Total RNA was extracted from tissues using Trizol reagent (Invitrogen, Carlsbad, CA, USA). RNA was purified and DNase treated using the RNeasy QIAGEN RNeasy^®^ Mini Kit (QIAGEN, Hilden, Germany). cDNA was synthesized from 2 μg of total-RNA using the direct cDNA Labeling System. Aminoallyl-cRNA was synthesized from cDNA using the Superscript Indirect cDNA Labeling System (Agilent, Palo Alto, CA, USA). The cRNA was purified and DNase treated using RNeasy QIAGEN RNeasy^®^ Mini Kit (QIAGEN, Hilden, Germany). The integrity of total RNA also passed analysis with the Bioanalyzer 2100 (model 2100; Agilent Technologies, Palo Alto, CA, USA) with RIN number > 6.0. Labeling and hybridization of the cRNA was performed with Agilent Whole Porcine Genome Oligo (4 × 44K) Microarrays (one-color platform) at the National Engineering Center for Biochip at Shanghai, according to the manufacturer’s protocols. The slides were scanned and analyzed using the histogram method with default settings in an Agilent G2565AA and Agilent G2565BA Microarray Scanner System using SureScan Technology. The TIFF image generated was loaded into Feature Extraction Software (Agilent Technologies, Sisha Clara, CA, USA) for feature data extraction, and data analysis was performed with MultiExperiment Viewer (MeV) (Boston, MA, USA). The array data has been submitted to gene expression omnibus (GEO) GSE42317 [50].

Comparisons between the CG and the TG were conducted using three biological replicates for each group. CG samples and TG samples were used for microarray analysis. The six Microarray data were normalized using the quantile method [51] with WebarrayDB online microarray data analysis [52]. Data were filtered and assessed by the MIDAW online analysis program [53] using the method of weighted K-nearest neighbor [54]. *T*-tests for microarray data were performed by a MultiExperiment Viewer (MeV) software package (Version 4.5, Dana-Farber Cancer Institute, 44 Binney St, Boston, MA, USA) [55].

Tests for statistical significance (*p* < 0.05), overrepresentation of Gene ontology (GO) terms [56,57], and pathway in Kyoto Encyclopedia of Genes and Genomes (KEGG) DB [58,59] among both induced and repressed genes were conducted using the ErmineJ gene set analysis software [60] and the Database for Annotation, Visualization and Integrated Discovery (DAVID) Online platform with a threshold of a minimum three genes annotated at each node. The leading edge analysis for the pathway of differential expression in microarray data with a threshold of a minimum of two genes and maximum of 20 genes annotated at each node were conducted using the gene set enrichment analysis (GSEA) V2.06 package at the GSEA/MSigDB web site (the broad institute) [61–63]. More detailed descriptions of the microarray experiments are available at the NCBI Gene Expression Omnibus [64–66].

### 4.3. Real-Time QRT-PCR

In order to confirm reliability of the expression profile in the microarray analyses, expression level of the genes (six up-regulated and four down-regulated) were performed by real-time qRT-PCR. Sequences for the primers were obtained from Genbank and NCBI. Primers were designed using the software Primer 5 and synthesized at Invitrogen Co. Led (Shanghai, China) (Table 2). Extracted RNA was converted into cDNA by reverse transcription of 1 μL total RNA using SYBR^®^ PrimeScript™ RT-PCR Kit (TaKaRa, Shiga, Japan) according to the manufacturer’s protocol, and then cDNA was stored at −20 °C until use. Quantitative PCR was performed in a 25 μL reaction volume (2 μL cDNA, 12.5 μL of SYBR^®^ Premix Ex TaqTM (2×) TaKaRa, Shiga, Japan), 0.5 μL of 10 μM upstream and downstream primers respectively, and added ddH_2_O to 25 μL) on the BIO-RAD IQ5 System (BIO-RAD, Hercules, CA, USA). Real-time PCR conditions were as follows: 30 s at 95.0 °C, 40 cycles of denaturation at 95 °C for 5 s followed by 30 s annealing and elongation at 51.2–60 °C (Table 2). Information of primer pairs is reported in Table 2. Melting curves were obtained at the end of each run to confirm a single PCR product. All samples were run in triplicate. Non-template controls were included in each run to exclude contamination and nonspecific amplification. Expression levels of samples were normalized by using a normalization factor calculated by the program geNorm. This normalization factor was calculated based on RT-qPCR results for three selected reference genes, *ACTB*, *TOP2B* and *TBP*.

This allowed quantification of the target gene in one sample relative to that in another (the calibrator) using the “2^−ΔΔ^*^C^*^t^ method” of calculating fold changes in gene expression [67]. Correlation analysis between qRT-PCR and microarray was conducted.

## 5. Conclusions

We have generated reliable mRNA transcriptomes of HN from the APP-infected and negative control pigs and identified a set of DE genes in our current case-control study, and a functional enrichment analysis indicated that these DE genes were mainly related to “host immune response” and “host response”. We found that, in the APP-infected HN, many cytokineswere activated and involved in cell migration signaling induced response cells as granulocytes (e.g., neutrophils, eosinophils, and basophils) and monocytes migration to the inflammatory site, while the cytokines involved in regulation of the host immune response were suppressed. The current study provides data that can be used in future studies to decipher the molecular mechanism of the systematic influences from porcine pleuropneumonia.

## Supplementary Information



## Figures and Tables

**Table 1.** Eigenvalues and contribution ratio of PCA for differential expression genes.

Principal Component	Eigenvalues	Contribution ratio
1	42.273	97.16%
2	0.978	2.25%
3	0.147	0.34%
4	0.072	0.17%
5	0.025	0.06%
6	0.014	0.03%

**Table 2.** Information on the primers used for QRT-PCR.

Confirmation objects	Gene symbol	Primer sequence (5′→3′)	Amplicon length (bp)	Ta (°C)	GenBank No.
Reference gene	*ACTB*	TCTGGCACCACACCTTCT	114	60	DQ178122
		TGATCTGGGTCATCTTCTCAC			

	*TBP*	GATGGACGTTCGGTTTAGG	124	60	DQ178129
		AGCAGCACAGTACGAGCAA			

	*TOP2B*	AACTGGATGATGCTAATGATGCT	137	60	AF222921
		TGGAAAAACTCCGTATCTGTCTC			

Up gene	*RETN*	AGTGCGCTGGCATAGACTGG	197	60	NM_213783
		CATCCTCTTCTCAAGGTTTATTTCC			

	*ADAM17*	TTGAGGAAGGGGAAGCC	158	56	NM_001099926
		ACGGAGCCCACGATGTT			

	*GPNMB*	GAGACCCAGCCTTCCTT	130	51.2	NM_001098584
		TTGCTTTCTATCGCTTTGTA			

	*CHRM1*	CGCTGGTCAAGGAGAAGAA	185	56	NM_214034
		GCACATGGGGTTGATGGT			

	*ALDH2*	AAACTGCTCTGCGGTGGA	181	56	NM_001044611
		CGTACTTGGAATTGTTGGCTC			

	*IL6*	GTCGAGGCTGTGCAGATTAG	101	56	NM_214399
		GCATTTGTGGTGGGGTTAG			

Down gene	*KLRK1*	TGATGTGATAAACCGTGGTG	107	56	NM_213813
		TGGATCGGGCAAGGAAA			

	*DUOX2*	CCCTTCTTCAACTCCCTG	158	51.2	NM_213999
		CAAAAGTTCTCATAGTGGTGC			

	*OAS2*	GACACGGCTGAAGGTTT	291	51.2	NM_001031796
		TGGCACGTCCCAAGACT			

	*KCNAB1*	AAGGGAGAAAACAGCAAAAC	176	56	NM_001105294
		AACCTGAATGGCACCGA			
