# Supplementary material for: Transcriptional Profiling of Hilar Nodes from Pigs after Experimental Infection with Actinobacillus Pleuropneumoniae
Source: Int J Mol Sci. 2013 Nov 29;14(12):23516–32. doi: 10.3390/ijms141223516 (PMC3876060; doi:10.3390/ijms141223516)
Supplement: Supplementary file 1 [file ijms-14-23516-s001.pdf]

Supplementary Information

Table S1. The significant gene ontology biological processes in swines.

Name	Describtion	Probe	Genes
[GO:0019538]	protein metabolic process	92	92
[GO:0009058]	biosynthetic process	80	80
[GO:0051179]	localization	73	73
[GO:0044249]	cellular biosynthetic process	73	73
[GO:0006810]	transport	70	70
[GO:0044267]	cellular protein metabolic process	67	67
[GO:0023052]	signaling	63	63
[GO:0006807]	nitrogen compound metabolic process	58	58
[GO:0034641]	cellular nitrogen compound metabolic process	55	55
[GO:0023060]	signal transmission	53	53
[GO:0007165]	signal transduction	51	51
[GO:0019222]	regulation of metabolic process	50	50
[GO:0006139]	cellular nucleobase, nucleoside, nucleotide and nucleic acid metabolic process	48	48
[GO:0044281]	small molecule metabolic process	48	48
[GO:0010467]	gene expression	48	48
[GO:0050896]	response to stimulus	47	47
[GO:0080090]	regulation of primary metabolic process	46	46
[GO:0031323]	regulation of cellular metabolic process	46	46
[GO:0060255]	regulation of macromolecule metabolic process	46	46
[GO:0055114]	oxidation reduction	45	45
[GO:0006464]	protein modification process	44	44
[GO:0034645]	cellular macromolecule biosynthetic process	43	43
[GO:0009889]	regulation of biosynthetic process	42	42
[GO:0010468]	regulation of gene expression	42	42
[GO:0051171]	regulation of nitrogen compound metabolic process	41	41
[GO:0009056]	catabolic process	41	41
[GO:0010556]	regulation of macromolecule biosynthetic process	41	41
[GO:0061019]	regulation of transcription	40	40
[GO:0006508]	proteolysis	38	38
[GO:0043687]	post-translational protein modification	36	36
[GO:0044248]	cellular catabolic process	34	34
[GO:0006355]	regulation of cellular transcription, DNA-dependent	33	33
[GO:0032502]	developmental process	33	33
[GO:0032501]	multicellular organismal process	33	33
[GO:0007275]	multicellular organismal development	31	31
[GO:0006796]	phosphate metabolic process	31	31
[GO:0006629]	lipid metabolic process	30	30
[GO:0016310]	phosphorylation	28	28
[GO:0016043]	cellular component organization	27	27
[GO:0005975]	carbohydrate metabolic process	27	27
[GO:0006950]	response to stress	27	27
[GO:0061018]	transcription	27	27

Table S1. *Cont.*

Name	Describtion	Probe	Genes
[GO:0006811]	ion transport	27	27
[GO:0002376]	immune system process	26	26
[GO:0006082]	organic acid metabolic process	26	26
[GO:0023033]	signaling pathway	26	26
[GO:0065008]	regulation of biological quality	25	25
[GO:0044283]	small molecule biosynthetic process	24	24
[GO:0007242]	intracellular signaling cascade	24	24
[GO:0006468]	protein amino acid phosphorylation	23	23
[GO:0044255]	cellular lipid metabolic process	22	22
[GO:0006955]	immune response	22	22
[GO:0007166]	cell surface receptor linked signaling pathway	22	22
[GO:0048856]	anatomical structure development	21	21
[GO:0009057]	macromolecule catabolic process	21	21
[GO:0032787]	monocarboxylic acid metabolic process	21	21
[GO:0033036]	macromolecule localization	20	20
[GO:0048869]	cellular developmental process	20	20
[GO:0048731]	system development	19	19
[GO:0006091]	generation of precursor metabolites and energy	19	19
[GO:0006812]	cation transport	19	19
[GO:0044265]	cellular macromolecule catabolic process	18	18
[GO:0044262]	cellular carbohydrate metabolic process	18	18
[GO:0030154]	cell differentiation	18	18
[GO:0006996]	organelle organization	18	18
[GO:0048518]	positive regulation of biological process	18	18
[GO:0048519]	negative regulation of biological process	18	18
[GO:0008219]	cell death	17	17
[GO:0048523]	negative regulation of cellular process	17	17
[GO:0006631]	fatty acid metabolic process	15	15
[GO:0010941]	regulation of cell death	15	15
[GO:0042592]	homeostatic process	15	15
[GO:0012501]	programmed cell death	15	15
[GO:0051186]	cofactor metabolic process	14	14
[GO:0044271]	cellular nitrogen compound biosynthetic process	14	14
[GO:0030163]	protein catabolic process	14	14
[GO:0006066]	alcohol metabolic process	14	14
[GO:0023034]	intracellular signaling pathway	14	14
[GO:0044257]	cellular protein catabolic process	13	13
[GO:0006952]	defense response	13	13
[GO:0048513]	organ development	13	13
[GO:0030001]	metal ion transport	13	13
[GO:0055086]	cellular nucleobase, nucleoside and nucleotide metabolic process	12	12
[GO:0007186]	G-protein coupled receptor protein signaling pathway	12	12
[GO:0043632]	modification-dependent macromolecule catabolic process	11	11
[GO:0008610]	lipid biosynthetic process	11	11

Table S1. *Cont.*

Name	Describtion	Probe	Genes
[GO:0008104]	protein localization	11	11
[GO:0046483]	heterocycle metabolic process	11	11
[GO:0015672]	monovalent inorganic cation transport	11	11
[GO:0007155]	cell adhesion	11	11
[GO:0048583]	regulation of response to stimulus	10	10
[GO:0044282]	small molecule catabolic process	10	10
[GO:0051716]	cellular response to stimulus	10	10
[GO:0042221]	response to chemical stimulus	10	10
[GO:0051704]	multi-organism process	10	10
[GO:0007049]	cell cycle	10	10
[GO:0045184]	establishment of protein localization	10	10
[GO:0048522]	positive regulation of cellular process	10	10
[GO:0015980]	energy derivation by oxidation of organic compounds	10	10
[GO:0009607]	response to biotic stimulus	9	9
[GO:0045333]	cellular respiration	9	9
[GO:0006412]	translation	9	9
[GO:0007264]	small GTPase mediated signal transduction	9	9
[GO:0019725]	cellular homeostasis	9	9
[GO:0046394]	carboxylic acid biosynthetic process	9	9
[GO:0009165]	nucleotide biosynthetic process	9	9
[GO:0000003]	reproduction	9	9
[GO:0009100]	glycoprotein metabolic process	8	8
[GO:0009109]	coenzyme catabolic process	8	8
[GO:0048584]	positive regulation of response to stimulus	8	8
[GO:0032879]	regulation of localization	8	8
[GO:0002682]	regulation of immune system process	8	8
[GO:0010876]	lipid localization	8	8
[GO:0019318]	hexose metabolic process	8	8
[GO:0009308]	amine metabolic process	8	8
[GO:0022414]	reproductive process	8	8
[GO:0008202]	steroid metabolic process	8	8
[GO:0009653]	anatomical structure morphogenesis	8	8
[GO:0019882]	antigen processing and presentation	8	8
[GO:0033554]	cellular response to stress	8	8
[GO:0009101]	glycoprotein biosynthetic process	7	7
[GO:0016042]	lipid catabolic process	7	7
[GO:0044085]	cellular component biogenesis	7	7
[GO:0002684]	positive regulation of immune system process	7	7
[GO:0006633]	fatty acid biosynthetic process	7	7
[GO:0016192]	vesicle-mediated transport	7	7
[GO:0009611]	response to wounding	7	7
[GO:0051301]	cell division	7	7
[GO:0031325]	positive regulation of cellular metabolic process	7	7
[GO:0006519]	cellular amino acid and derivative metabolic process	7	7

Table S1. *Cont.*

Name	Describtion	Probe	Genes
[GO:0010033]	response to organic substance	7	7
[GO:0051128]	regulation of cellular component organization	7	7
[GO:0006163]	purine nucleotide metabolic process	7	7
[GO:0051641]	cellular localization	7	7
[GO:0051276]	chromosome organization	7	7
[GO:0051173]	positive regulation of nitrogen compound metabolic process	6	6
[GO:0006325]	chromatin organization	6	6
[GO:0002526]	acute inflammatory response	6	6
[GO:0045454]	cell redox homeostasis	6	6
[GO:0046907]	intracellular transport	6	6
[GO:0070887]	cellular response to chemical stimulus	6	6
[GO:0050776]	regulation of immune response	6	6
[GO:0051707]	response to other organism	6	6
[GO:0032535]	regulation of cellular component size	6	6
[GO:0002252]	immune effector process	6	6
[GO:0051605]	protein maturation by peptide bond cleavage	6	6
[GO:0043069]	negative regulation of programmed cell death	6	6
[GO:0006820]	anion transport	6	6
[GO:0051239]	regulation of multicellular organismal process	6	6
[GO:0009260]	ribonucleotide biosynthetic process	6	6
[GO:0016070]	cellular RNA metabolic process	6	6
[GO:0065009]	regulation of molecular function	6	6
[GO:0006869]	lipid transport	6	6
[GO:0007167]	enzyme linked receptor protein signaling pathway	6	6
[GO:0008643]	carbohydrate transport	5	5
[GO:0016051]	carbohydrate biosynthetic process	5	5
[GO:0016052]	carbohydrate catabolic process	5	5
[GO:0042127]	regulation of cell proliferation	5	5
[GO:0019953]	sexual reproduction	5	5
[GO:0006644]	phospholipid metabolic process	5	5
[GO:0040008]	regulation of growth	5	5
[GO:0055085]	transmembrane transport	5	5
[GO:0007399]	nervous system development	5	5
[GO:0050790]	regulation of catalytic activity	5	5
[GO:0050793]	regulation of developmental process	5	5
[GO:0022900]	electron transport chain	5	5
[GO:0016568]	chromatin modification	5	5
[GO:0007243]	protein kinase cascade	5	5
[GO:0009888]	tissue development	5	5
[GO:0048878]	chemical homeostasis	5	5
[GO:0050778]	positive regulation of immune response	5	5
[GO:0010604]	positive regulation of macromolecule metabolic process	5	5
[GO:0044106]	cellular amine metabolic process	5	5
[GO:0006357]	regulation of transcription from RNA polymerase II promoter	5	5

Table S1. *Cont.*

Name	Describtion	Probe	Genes
[GO:0006259]	cellular DNA metabolic process	5	5
[GO:0016125]	sterol metabolic process	5	5
[GO:0031328]	positive regulation of cellular biosynthetic process	5	5
[GO:0006090]	pyruvate metabolic process	5	5
[GO:0007010]	cytoskeleton organization	5	5
[GO:0006814]	sodium ion transport	5	5
[GO:0009199]	ribonucleoside triphosphate metabolic process	5	5
[GO:0051129]	negative regulation of cellular component organization	5	5
[GO:0006694]	steroid biosynthetic process	5	5
[GO:0006886]	intracellular protein transport	5	5
[GO:0051246]	regulation of protein metabolic process	5	5
[GO:0016071]	mRNA metabolic process	5	5
[GO:0065003]	macromolecular complex assembly	5	5
[GO:0006119]	oxidative phosphorylation	4	4
[GO:0048646]	anatomical structure formation involved in morphogenesis	4	4
[GO:0009108]	coenzyme biosynthetic process	4	4
[GO:0002520]	immune system development	4	4
[GO:0034984]	cellular response to DNA damage stimulus	4	4
[GO:0001501]	skeletal system development	4	4
[GO:0009628]	response to abiotic stimulus	4	4
[GO:0033043]	regulation of organelle organization	4	4
[GO:0040007]	growth	4	4
[GO:0044087]	regulation of cellular component biogenesis	4	4
[GO:0002504]	antigen processing and presentation of peptide or polysaccharide antigen via MHC class II	4	4
[GO:0008361]	regulation of cell size	4	4
[GO:0006511]	ubiquitin-dependent protein catabolic process	4	4
[GO:0006800]	oxygen and reactive oxygen species metabolic process	4	4
[GO:0048609]	reproductive process in a multicellular organism	4	4
[GO:0010605]	negative regulation of macromolecule metabolic process	4	4
[GO:0008380]	RNA splicing	4	4
[GO:0006725]	cellular aromatic compound metabolic process	4	4
[GO:0051049]	regulation of transport	4	4
[GO:0000087]	M phase of mitotic cell cycle	4	4
[GO:0008203]	cholesterol metabolic process	4	4
[GO:0006520]	cellular amino acid metabolic process	4	4
[GO:0006916]	anti-apoptosis	4	4
[GO:0016126]	sterol biosynthetic process	4	4
[GO:0006094]	gluconeogenesis	4	4
[GO:0030155]	regulation of cell adhesion	4	4
[GO:0006096]	glycolysis	4	4
[GO:0006457]	protein folding	4	4
[GO:0030258]	lipid modification	4	4
[GO:0071310]	cellular response to organic substance	4	4

Table S1. *Cont.*

Name	Describtion	Probe	Genes
[GO:0051336]	regulation of hydrolase activity	4	4
[GO:0080134]	regulation of response to stress	4	4
[GO:0006575]	cellular amino acid derivative metabolic process	4	4
[GO:0006818]	hydrogen transport	4	4
[GO:0051726]	regulation of cell cycle	4	4
[GO:0061024]	membrane organization	4	4
[GO:0061020]	positive regulation of transcription	4	4
[GO:0015674]	di-, tri-valent inorganic cation transport	4	4
[GO:0046395]	carboxylic acid catabolic process	4	4
[GO:0008284]	positive regulation of cell proliferation	4	4
[GO:0034621]	cellular macromolecular complex subunit organization	4	4
[GO:0010646]	regulation of cell communication	4	4
[GO:0006650]	glycerophospholipid metabolic process	3	3
[GO:0001503]	ossification	3	3
[GO:0016049]	cell growth	3	3
[GO:0009719]	response to endogenous stimulus	3	3
[GO:0048872]	homeostasis of number of cells	3	3
[GO:0009617]	response to bacterium	3	3
[GO:0045137]	development of primary sexual characteristics	3	3
[GO:0009615]	response to virus	3	3
[GO:0055082]	cellular chemical homeostasis	3	3
[GO:0009116]	nucleoside metabolic process	3	3
[GO:0006801]	superoxide metabolic process	3	3
[GO:0007610]	behavior	3	3
[GO:0045884]	regulation of survival gene product expression	3	3
[GO:0031347]	regulation of defense response	3	3
[GO:0031333]	negative regulation of protein complex assembly	3	3
[GO:0007259]	JAK-STAT cascade	3	3
[GO:0031324]	negative regulation of cellular metabolic process	3	3
[GO:0032989]	cellular component morphogenesis	3	3
[GO:0005976]	polysaccharide metabolic process	3	3
[GO:0043281]	regulation of caspase activity	3	3
[GO:0048511]	rhythmic process	3	3
[GO:0009062]	fatty acid catabolic process	3	3
[GO:0006816]	calcium ion transport	3	3
[GO:0007017]	microtubule-based process	3	3
[GO:0001568]	blood vessel development	3	3
[GO:0007346]	regulation of mitotic cell cycle	3	3
[GO:0042325]	regulation of phosphorylation	3	3
[GO:0006790]	sulfur metabolic process	3	3
[GO:0006695]	cholesterol biosynthetic process	3	3
[GO:0019221]	cytokine-mediated signaling pathway	3	3
[GO:0001558]	regulation of cell growth	3	3

Table S1. *Cont.*

Name	Describtion	Probe	Genes
[GO:0007178]	transmembrane receptor protein serine/threonine kinase signaling pathway	3	3
[GO:0006979]	response to oxidative stress	3	3
[GO:0045927]	positive regulation of growth	3	3
[GO:0015985]	energy coupled proton transport, down electrochemical gradient	3	3
[GO:0019216]	regulation of lipid metabolic process	3	3
[GO:0006897]	endocytosis	3	3
[GO:0007154]	cell communication	3	3
[GO:0006470]	protein amino acid dephosphorylation	3	3
[GO:0045944]	positive regulation of transcription from RNA polymerase II promoter	3	3
[GO:0006461]	protein complex assembly	3	3
[GO:0007169]	transmembrane receptor protein tyrosine kinase signaling pathway	3	3
[GO:0051270]	regulation of cellular component movement	3	3

Table S2. The significant gene ontology cellular components in swines.

Name	Description	Probe	Genes
[GO:0044422]	organelle part	69	69
[GO:0005634]	nucleus	68	68
[GO:0044446]	intracellular organelle part	68	68
[GO:0032991]	macromolecular complex	62	62
[GO:0005886]	plasma membrane	60	60
[GO:0005576]	extracellular region	52	52
[GO:0043234]	protein complex	49	49
[GO:0005739]	mitochondrion	36	36
[GO:0005783]	endoplasmic reticulum	34	34
[GO:0043228]	non-membrane-bounded organelle	32	32
[GO:0044459]	plasma membrane part	30	30
[GO:0031090]	organelle membrane	30	30
[GO:0005794]	Golgi apparatus	28	28
[GO:0044429]	mitochondrial part	23	23
[GO:0044421]	extracellular region part	22	22
[GO:0031974]	membrane-enclosed lumen	21	21
[GO:0005856]	cytoskeleton	20	20
[GO:0012505]	endomembrane system	17	17
[GO:0031975]	envelope	17	17
[GO:0031982]	vesicle	15	15
[GO:0044430]	cytoskeletal part	14	14
[GO:0044428]	nuclear part	14	14
[GO:0016023]	cytoplasmic membrane-bounded vesicle	13	13
[GO:0005740]	mitochondrial envelope	13	13
[GO:0005829]	cytosol	12	12
[GO:0031966]	mitochondrial membrane	12	12
[GO:0071212]	subsynaptic reticulum	11	11
[GO:0005759]	mitochondrial matrix	11	11

Table S2. *Cont.*

Name	Description	Probe	Genes
[GO:0019898]	extrinsic to membrane	10	10
[GO:0015630]	microtubule cytoskeleton	10	10
[GO:0031012]	extracellular matrix	10	10
[GO:0030529]	ribonucleoprotein complex	10	10
[GO:0005773]	vacuole	9	9
[GO:0005578]	proteinaceous extracellular matrix	9	9
[GO:0009898]	internal side of plasma membrane	9	9
[GO:0005792]	microsome	8	8
[GO:0048770]	pigment granule	8	8
[GO:0005764]	lysosome	8	8
[GO:0044432]	endoplasmic reticulum part	8	8
[GO:0044431]	Golgi apparatus part	8	8
[GO:0031981]	nuclear lumen	8	8
[GO:0042611]	MHC protein complex	7	7
[GO:0005840]	ribosome	7	7
[GO:0000502]	proteasome complex	7	7
[GO:0005743]	mitochondrial inner membrane	7	7
[GO:0000139]	Golgi membrane	7	7
[GO:0005789]	endoplasmic reticulum membrane	6	6
[GO:0031225]	anchored to membrane	6	6
[GO:0005654]	nucleoplasm	6	6
[GO:0005874]	microtubule	6	6
[GO:0005819]	spindle	5	5
[GO:0042613]	MHC class II protein complex	5	5
[GO:0031968]	organelle outer membrane	5	5
[GO:0044451]	nucleoplasm part	5	5
[GO:0031300]	intrinsic to organelle membrane	4	4
[GO:0005887]	integral to plasma membrane	4	4
[GO:0005635]	nuclear envelope	4	4
[GO:0005911]	cell-cell junction	4	4
[GO:0005777]	peroxisome	4	4
[GO:0005768]	endosome	4	4
[GO:0046930]	pore complex	4	4
[GO:0015629]	actin cytoskeleton	4	4
[GO:0016469]	proton-transporting two-sector ATPase complex	4	4
[GO:0005694]	chromosome	3	3
[GO:0031301]	integral to organelle membrane	3	3
[GO:0048471]	perinuclear region of cytoplasm	3	3
[GO:0043292]	contractile fiber	3	3
[GO:0045202]	synapse	3	3
[GO:0031965]	nuclear membrane	3	3
[GO:0016323]	basolateral plasma membrane	3	3
[GO:0005681]	spliceosomal complex	3	3
[GO:0033176]	proton-transporting V-type ATPase complex	3	3
[GO:0005730]	nucleolus	3	3
[GO:0016604]	nuclear body	3	3
[GO:0022624]	proteasome accessory complex	3	3

Table S3. The significant gene ontology molecular functions in swines.

Name	Describtion	Probe	Genes
[GO:0000166]	nucleotide binding	96	96
[GO:0017076]	purine nucleotide binding	78	78
[GO:0016787]	hydrolase activity	73	73
[GO:0046914]	transition metal ion binding	70	70
[GO:0032555]	purine ribonucleotide binding	70	70
[GO:0016740]	transferase activity	68	68
[GO:0030554]	adenyl nucleotide binding	61	61
[GO:0003676]	nucleic acid binding	56	56
[GO:0032559]	adenyl ribonucleotide binding	53	53
[GO:0005524]	ATP binding	52	52
[GO:0016491]	oxidoreductase activity	50	50
[GO:0005215]	transporter activity	50	50
[GO:0060089]	molecular transducer activity	49	49
[GO:0008270]	zinc ion binding	43	43
[GO:0022892]	substrate-specific transporter activity	39	39
[GO:0004872]	receptor activity	37	37
[GO:0022857]	transmembrane transporter activity	35	35
[GO:0030528]	transcription regulator activity	34	34
[GO:0016772]	transferase activity, transferring phosphorus-containing groups	34	34
[GO:0003677]	DNA binding	34	34
[GO:0022891]	substrate-specific transmembrane transporter activity	33	33
[GO:0016301]	kinase activity	30	30
[GO:0015075]	ion transmembrane transporter activity	30	30
[GO:0008233]	peptidase activity	29	29
[GO:0016773]	phosphotransferase activity, alcohol group as acceptor	27	27
[GO:0005102]	receptor binding	26	26
[GO:0070011]	peptidase activity, acting on L-amino acid peptides	25	25
[GO:0048037]	cofactor binding	25	25
[GO:0003700]	transcription factor activity	25	25
[GO:0004672]	protein kinase activity	24	24
[GO:0004175]	endopeptidase activity	22	22
[GO:0008324]	cation transmembrane transporter activity	21	21
[GO:0005509]	calcium ion binding	21	21
[GO:0005506]	iron ion binding	21	21
[GO:0050662]	coenzyme binding	20	20
[GO:0030234]	enzyme regulator activity	19	19
[GO:0019001]	guanyl nucleotide binding	18	18
[GO:0016788]	hydrolase activity, acting on ester bonds	16	16
[GO:0009055]	electron carrier activity	16	16
[GO:0004888]	transmembrane receptor activity	16	16
[GO:0016817]	hydrolase activity, acting on acid anhydrides	15	15
[GO:0005198]	structural molecule activity	15	15
[GO:0004857]	enzyme inhibitor activity	15	15
[GO:0004674]	protein serine/threonine kinase activity	15	15
[GO:0022890]	inorganic cation transmembrane transporter activity	14	14

Table S3. *Cont.*

Name	Describtion	Probe	Genes
[GO:0000287]	magnesium ion binding	14	14
[GO:0022804]	active transmembrane transporter activity	13	13
[GO:0008092]	cytoskeletal protein binding	13	13
[GO:0030414]	peptidase inhibitor activity	12	12
[GO:0016757]	transferase activity, transferring glycosyl groups	12	12
[GO:0043565]	sequence-specific DNA binding	11	11
[GO:0017171]	serine hydrolase activity	11	11
[GO:0016746]	transferase activity, transferring acyl groups	11	11
[GO:0016614]	oxidoreductase activity, acting on CH-OH group of donors	11	11
[GO:0016874]	ligase activity	10	10
[GO:0016705]	oxidoreductase activity, acting on paired donors, with incorporation or reduction of molecular oxygen	10	10
[GO:0046906]	tetrapyrrole binding	9	9
[GO:0016887]	ATPase activity	9	9
[GO:0008289]	lipid binding	9	9
[GO:0008234]	cysteine-type peptidase activity	9	9
[GO:0004930]	G-protein coupled receptor activity	9	9
[GO:0050660]	FAD binding	8	8
[GO:0046873]	metal ion transmembrane transporter activity	8	8
[GO:0042623]	ATPase activity, coupled	8	8
[GO:0042578]	phosphoric ester hydrolase activity	8	8
[GO:0022838]	substrate-specific channel activity	8	8
[GO:0020037]	heme binding	8	8
[GO:0019842]	vitamin binding	8	8
[GO:0016747]	transferase activity, transferring acyl groups other than amino-acyl groups	8	8
[GO:0015078]	hydrogen ion transmembrane transporter activity	8	8
[GO:0005125]	cytokine activity	8	8
[GO:0004197]	cysteine-type endopeptidase activity	8	8
[GO:0003723]	RNA binding	8	8
[GO:0051287]	NAD or NADH binding	7	7
[GO:0046983]	protein dimerization activity	7	7
[GO:0030246]	carbohydrate binding	7	7
[GO:0016791]	phosphatase activity	7	7
[GO:0016758]	transferase activity, transferring hexosyl groups	7	7
[GO:0015291]	secondary active transmembrane transporter activity	7	7
[GO:0008083]	growth factor activity	7	7
[GO:0005126]	cytokine receptor binding	7	7
[GO:0004713]	protein tyrosine kinase activity	7	7
[GO:0003779]	actin binding	7	7
[GO:0003735]	structural constituent of ribosome	7	7
[GO:0031406]	carboxylic acid binding	6	6
[GO:0030145]	manganese ion binding	6	6
[GO:0022836]	gated channel activity	6	6
[GO:0016879]	ligase activity, forming carbon-nitrogen bonds	6	6

Table S3. *Cont.*

Name	Describtion	Probe	Genes
[GO:0016810]	hydrolase activity, acting on carbon-nitrogen (but not peptide) bonds	6	6
[GO:0016209]	antioxidant activity	6	6
[GO:0008509]	anion transmembrane transporter activity	6	6
[GO:0008430]	selenium binding	6	6
[GO:0004721]	phosphoprotein phosphatase activity	6	6
[GO:0004497]	monooxygenase activity	6	6
[GO:0050661]	NADP or NADPH binding	5	5
[GO:0042626]	ATPase activity, coupled to transmembrane movement of substances	5	5
[GO:0031420]	alkali metal ion binding	5	5
[GO:0019904]	protein domain specific binding	5	5
[GO:0016853]	isomerase activity	5	5
[GO:0016811]	hydrolase activity, acting on carbon-nitrogen (but not peptide) bonds, in linear amides	5	5
[GO:0016798]	hydrolase activity, acting on glycosyl bonds	5	5
[GO:0016779]	nucleotidyltransferase activity	5	5
[GO:0016563]	transcription activator activity	5	5
[GO:0015293]	symporter activity	5	5
[GO:0015082]	di-, tri-valent inorganic cation transmembrane transporter activity	5	5
[GO:0005507]	copper ion binding	5	5
[GO:0004867]	serine-type endopeptidase inhibitor activity	5	5
[GO:0070403]	NAD binding	4	4
[GO:0070279]	vitamin B6 binding	4	4
[GO:0051540]	metal cluster binding	4	4
[GO:0046915]	transition metal ion transmembrane transporter activity	4	4
[GO:0042625]	ATPase activity, coupled to transmembrane movement of ions	4	4
[GO:0019955]	cytokine binding	4	4
[GO:0019899]	enzyme binding	4	4
[GO:0019199]	transmembrane receptor protein kinase activity	4	4
[GO:0016881]	acid-amino acid ligase activity	4	4
[GO:0016860]	intramolecular oxidoreductase activity	4	4
[GO:0016741]	transferase activity, transferring one-carbon groups	4	4
[GO:0016709]	oxidoreductase activity, acting on paired donors, with incorporation or reduction of molecular oxygen, NADH or NADPH as one donor, and incorporation of one atom of oxygen	4	4
[GO:0016684]	oxidoreductase activity, acting on peroxide as acceptor	4	4
[GO:0016627]	oxidoreductase activity, acting on the CH-CH group of donors	4	4
[GO:0015631]	tubulin binding	4	4
[GO:0008134]	transcription factor binding	4	4
[GO:0005529]	sugar binding	4	4
[GO:0005516]	calmodulin binding	4	4
[GO:0005253]	anion channel activity	4	4
[GO:0004869]	cysteine-type endopeptidase inhibitor activity	4	4
[GO:0004386]	helicase activity	4	4
[GO:0004091]	carboxylesterase activity	4	4
[GO:0001664]	G-protein-coupled receptor binding	4	4

Table S3. *Cont.*

Name	Describtion	Probe	Genes
[GO:0060589]	nucleoside-triphosphatase regulator activity	3	3
[GO:0051427]	hormone receptor binding	3	3
[GO:0046943]	carboxylic acid transmembrane transporter activity	3	3
[GO:0046912]	transferase activity, transferring acyl groups, acyl groups converted into alkyl on transfer	3	3
[GO:0042803]	protein homodimerization activity	3	3
[GO:0031402]	sodium ion binding	3	3
[GO:0022843]	voltage-gated cation channel activity	3	3
[GO:0019838]	growth factor binding	3	3
[GO:0019829]	cation-transporting ATPase activity	3	3
[GO:0019787]	small conjugating protein ligase activity	3	3
[GO:0016829]	lyase activity	3	3
[GO:0016790]	thiolester hydrolase activity	3	3
[GO:0016769]	transferase activity, transferring nitrogenous groups	3	3
[GO:0016712]	oxidoreductase activity, acting on paired donors, with incorporation or reduction of molecular oxygen, reduced flavin or flavoprotein as one donor, and incorporation of one atom of oxygen	3	3
[GO:0016702]	oxidoreductase activity, acting on single donors with incorporation of molecular oxygen, incorporation of two atoms of oxygen	3	3
[GO:0016675]	oxidoreductase activity, acting on heme group of donors	3	3
[GO:0016667]	oxidoreductase activity, acting on sulfur group of donors	3	3
[GO:0016651]	oxidoreductase activity, acting on NADH or NADPH	3	3
[GO:0016407]	acetyltransferase activity	3	3
[GO:0016298]	lipase activity	3	3
[GO:0015929]	hexosaminidase activity	3	3
[GO:0015294]	solute:cation symporter activity	3	3
[GO:0015144]	carbohydrate transmembrane transporter activity	3	3
[GO:0010860]	proteasome regulator activity	3	3
[GO:0008565]	protein transporter activity	3	3
[GO:0008528]	peptide receptor activity, G-protein coupled	3	3
[GO:0008308]	voltage-gated anion channel activity	3	3
[GO:0008170]	N-methyltransferase activity	3	3
[GO:0008026]	ATP-dependent helicase activity	3	3
[GO:0008009]	chemokine activity	3	3
[GO:0005539]	glycosaminoglycan binding	3	3
[GO:0005385]	zinc ion transmembrane transporter activity	3	3
[GO:0005179]	hormone activity	3	3
[GO:0004879]	ligand-dependent nuclear receptor activity	3	3
[GO:0004725]	protein tyrosine phosphatase activity	3	3
[GO:0004715]	non-membrane spanning protein tyrosine kinase activity	3	3
[GO:0004576]	oligosaccharyl transferase activity	3	3
[GO:0004198]	calcium-dependent cysteine-type endopeptidase activity	3	3
[GO:0003995]	acyl-CoA dehydrogenase activity	3	3
[GO:0003743]	translation initiation factor activity	3	3
[GO:0003712]	transcription cofactor activity	3	3

Table S4. Pathways enriched in phenotype TG.

Pathway	Size	ES	NES	NOM *p*-val	FDR *q*-val	FWER *p*-val	Rank At Max	Leading Edge
ssc04370:VEGF signaling pathway	6	−0.76	−1.62	0.009	1	0.774	46	tags = 67%, list = 13%, signal = 76%
ssc04722:Neurotrophin signaling pathway	5	−0.74	−1.54	0.029	1	0.95	49	tags = 60%, list = 14%, signal = 69%
ssc04610:Complement and coagulation cascades	9	−0.61	−1.5	0.054	1	0.981	24	tags = 44%, list = 7%, signal = 47%
ssc04660:T cell receptor signaling pathway	14	−0.53	−1.46	0.072	1	0.995	134	tags = 79%, list = 39%, signal = 123%
ssc00410:beta-Alanine metabolism	5	−0.68	−1.4	0.09	1	1	22	tags = 40%, list = 6%, signal = 42%
ssc05216:Thyroid cancer	6	−0.63	−1.39	0.118	1	1	35	tags = 33%, list = 10%, signal = 36%
ssc04810:Regulation of actin cytoskeleton	16	−0.48	−1.38	0.123	1	1	48	tags = 31%, list = 14%, signal = 35%
ssc04012:ErbB signaling pathway	16	−0.49	−1.37	0.105	1	1	17	tags = 19%, list = 5%, signal = 19%
ssc04330:Notch signaling pathway	5	−0.67	−1.37	0.108	1	1	61	tags = 60%, list = 18%, signal = 72%
ssc04080:Neuroactive ligand-receptor interaction	9	−0.55	−1.36	0.133	1	1	20	tags = 33%, list = 6%, signal = 34%
ssc00520:Amino sugar and nucleotide sugar metabolism	5	−0.65	−1.34	0.124	1	1	60	tags = 60%, list = 17%, signal = 72%
ssc04730:Long-term depression	18	−0.45	−1.34	0.132	1	1	77	tags = 44%, list = 22%, signal = 54%
ssc00640:Propanoate metabolism	9	−0.54	−1.32	0.155	1	1	42	tags = 33%, list = 12%, signal = 37%
ssc04664:Fc epsilon RI signaling pathway	8	−0.56	−1.31	0.144	1	1	132	tags = 75%, list = 38%, signal = 119%
ssc04912:GnRH signaling pathway	6	−0.61	−1.31	0.148	1	1	49	tags = 50%, list = 14%, signal = 57%
ssc04662:B cell receptor signaling pathway	12	−0.48	−1.3	0.18	1	1	134	tags = 67%, list = 39%, signal = 105%
ssc00650:Butanoate metabolism	6	−0.6	−1.29	0.181	1	1	121	tags = 83%, list = 35%, signal = 126%
ssc04621:NOD-like receptor signaling pathway	12	−0.49	−1.29	0.166	0.95	1	145	tags = 75%, list = 42%, signal = 125%
ssc00310:Lysine degradation	6	−0.59	−1.28	0.168	0.945	1	122	tags = 83%, list = 35%, signal = 127%
ssc05211:Renal cell carcinoma	8	−0.54	−1.27	0.188	0.927	1	132	tags = 75%, list = 38%, signal = 119%
ssc05221:Acute myeloid leukemia	14	−0.47	−1.27	0.175	0.904	1	132	tags = 71%, list = 38%, signal = 111%

Table S4. *Cont.*

**Pathway**	**Size**	**ES**	**NES**	**NOM *p*-val**	**FDR *q*-val**	**FWER *p*-val**	**Rank At Max**	**Leading Edge**
ssc05212:Pancreatic cancer	16	−0.45	−1.27	0.207	0.874	1	113	tags = 63%, list = 33%, signal = 89%
ssc04270:Vascular smooth muscle contraction	3	−0.71	−1.24	0.197	0.927	1	49	tags = 67%, list = 14%, signal = 77%
ssc00053:Ascorbate and aldarate metabolism	2	−0.77	−1.22	0.218	0.974	1	8	tags = 50%, list = 2%, signal = 51%
ssc05222:Small cell lung cancer	15	−0.44	−1.21	0.223	0.975	1	105	tags = 53%, list = 31%, signal = 73%
ssc04020:Calcium signaling pathway	19	−0.4	−1.21	0.244	0.94	1	52	tags = 26%, list = 15%, signal = 29%
ssc00260:Glycine, serine and threonine metabolism	2	−0.76	−1.21	0.193	0.917	1	85	tags = 100%, list = 25%, signal = 132%
ssc00620:Pyruvate metabolism	7	−0.54	−1.21	0.24	0.885	1	27	tags = 29%, list = 8%, signal = 30%
ssc04130:SNARE interactions in vesicular transport	13	−0.43	−1.19	0.251	0.939	1	164	tags = 77%, list = 48%, signal = 141%
ssc03050:Proteasome	7	−0.51	−1.18	0.283	0.92	1	82	tags = 43%, list = 24%, signal = 55%
ssc04114:Oocyte meiosis	9	−0.49	−1.18	0.28	0.902	1	49	tags = 33%, list = 14%, signal = 38%
ssc04640:Hematopoietic cell lineage	7	−0.52	−1.18	0.291	0.883	1	78	tags = 57%, list = 23%, signal = 72%
ssc00280:Valine, leucine and isoleucine degradation	12	−0.44	−1.18	0.279	0.859	1	121	tags = 58%, list = 35%, signal = 87%
ssc04914:Progesterone-mediated oocyte maturation	7	−0.52	−1.17	0.28	0.847	1	46	tags = 43%, list = 13%, signal = 48%
ssc00903:Limonene and pinene degradation	3	−0.66	−1.17	0.266	0.827	1	121	tags = 100%, list = 35%, signal = 153%
ssc00071:Fatty acid metabolism	9	−0.47	−1.17	0.293	0.816	1	188	tags = 100%, list = 55%, signal = 215%
ssc00380:Tryptophan metabolism	4	−0.61	−1.16	0.272	0.803	1	56	tags = 50%, list = 16%, signal = 59%
ssc04740:Olfactory transduction	2	−0.73	−1.16	0.267	0.786	1	49	tags = 50%, list = 14%, signal = 58%
ssc00564:Glycerophospholipid metabolism	5	−0.57	−1.16	0.313	0.778	1	114	tags = 80%, list = 33%, signal = 118%
ssc00561:Glycerolipid metabolism	5	−0.55	−1.16	0.312	0.76	1	8	tags = 20%, list = 2%, signal = 20%
ssc00980:Metabolism of xenobiotics by cytochrome P450	5	−0.55	−1.15	0.29	0.768	1	26	tags = 40%, list = 8%, signal = 43%

Table S4. *Cont.*

Pathway	Size	ES	NES	NOM *p*-val	FDR *q*-val	FWER *p*-val	Rank At Max	Leading Edge
ssc04916:Melanogenesis	8	−0.48	−1.14	0.309	0.759	1	134	tags = 63%, list = 39%, signal = 100%
ssc04630:Jak-STAT signaling pathway	15	−0.4	−1.13	0.325	0.777	1	80	tags = 53%, list = 23%, signal = 66%
ssc00350:Tyrosine metabolism	4	−0.59	−1.13	0.322	0.762	1	93	tags = 75%, list = 27%, signal = 102%
ssc04070:Phosphatidylinositol signaling system	6	−0.51	−1.12	0.326	0.759	1	49	tags = 50%, list = 14%, signal = 57%
ssc00603:Glycosphingolipid biosynthesis	4	−0.58	−1.12	0.337	0.761	1	41	tags = 50%, list = 12%, signal = 56%
ssc04650:Natural killer cell mediated cytotoxicity	14	−0.4	−1.12	0.339	0.748	1	132	tags = 57%, list = 38%, signal = 89%
ssc00140:Steroid hormone biosynthesis	2	−0.71	−1.11	0.346	0.741	1	16	tags = 50%, list = 5%, signal = 52%
ssc00600:Sphingolipid metabolism	2	−0.7	−1.1	0.346	0.746	1	107	tags = 100%, list = 31%, signal = 144%
ssc03040:Spliceosome	8	−0.48	−1.1	0.368	0.749	1	109	tags = 50%, list = 32%, signal = 71%
ssc00010:Glycolysis/ Gluconeogenesis	9	−0.45	−1.09	0.385	0.744	1	67	tags = 33%, list = 19%, signal = 40%
ssc00190:Oxidative phosphorylation	11	−0.43	−1.09	0.363	0.745	1	94	tags = 36%, list = 27%, signal = 48%
ssc04530:Tight junction	10	−0.43	−1.08	0.385	0.744	1	23	tags = 20%, list = 7%, signal = 21%
ssc01040:Biosynthesis of unsaturated fatty acids	2	−0.67	−1.08	0.367	0.737	1	116	tags = 100%, list = 34%, signal = 150%
ssc00450:Selenoamino acid metabolism	3	−0.6	−1.08	0.379	0.727	1	139	tags = 100%, list = 40%, signal = 166%
ssc04210:Apoptosis	20	−0.34	−1.03	0.445	0.811	1	184	tags = 70%, list = 53%, signal = 142%
ssc00900:Terpenoid backbone biosynthesis	2	−0.65	−1.03	0.442	0.803	1	124	tags = 100%, list = 36%, signal = 155%
ssc00330:Arginine and proline metabolism	7	−0.44	−1.02	0.441	0.804	1	131	tags = 71%, list = 38%, signal = 113%
ssc00360:Phenylalanine metabolism	5	−0.5	−1.01	0.446	0.814	1	93	tags = 60%, list = 27%, signal = 81%
ssc04144:Endocytosis	13	−0.37	−1	0.491	0.831	1	19	tags = 15%, list = 6%, signal = 16%
ssc05020:Prion diseases	5	−0.49	−1	0.49	0.822	1	25	tags = 40%, list = 7%, signal = 43%
ssc04540:Gap junction	8	−0.42	−1	0.48	0.81	1	20	tags = 25%, list = 6%, signal = 26%

Table S4. *Cont.*

Pathway	Size	ES	NES	NOM *p*-val	FDR *q*-val	FWER *p*-val	Rank At Max	Leading Edge
ssc00020:Citrate cycle (TCA cycle)	11	−0.37	−0.97	0.502	0.858	1	221	tags = 100%, list = 64%, signal = 271%
ssc05214:Glioma	14	−0.35	−0.97	0.513	0.845	1	49	tags = 29%, list = 14%, signal = 32%
ssc05223:Non-small cell lung cancer	9	−0.4	−0.97	0.522	0.834	1	45	tags = 33%, list = 13%, signal = 37%
ssc00604:Glycosphingolipid biosynthesis	2	−0.61	−0.97	0.538	0.823	1	135	tags = 100%, list = 39%, signal = 164%
ssc05210:Colorectal cancer	13	−0.35	−0.96	0.509	0.814	1	134	tags = 54%, list = 39%, signal = 85%
ssc00500:Starch and sucrose metabolism	4	−0.51	−0.96	0.544	0.818	1	60	tags = 50%, list = 17%, signal = 60%
ssc00511:Other glycan degradation	2	−0.61	−0.95	0.568	0.81	1	135	tags = 100%, list = 39%, signal = 164%
ssc04360:Axon guidance	7	−0.43	−0.95	0.549	0.799	1	148	tags = 71%, list = 43%, signal = 123%
ssc04623:Cytosolic DNA-sensing pathway	9	−0.38	−0.94	0.542	0.808	1	105	tags = 44%, list = 31%, signal = 62%
ssc00040:Pentose and glucuronate interconversions	3	−0.55	−0.94	0.561	0.801	1	159	tags = 100%, list = 46%, signal = 184%
ssc04920:Adipocytokine signaling pathway	15	−0.34	−0.94	0.538	0.792	1	140	tags = 73%, list = 41%, signal = 118%
ssc00340:Histidine metabolism	4	−0.48	−0.93	0.569	0.802	1	56	tags = 50%, list = 16%, signal = 59%
ssc00591:Linoleic acid metabolism	2	−0.6	−0.93	0.583	0.791	1	16	tags = 50%, list = 5%, signal = 52%
ssc05213:Endometrial cancer	12	−0.35	−0.93	0.565	0.785	1	45	tags = 25%, list = 13%, signal = 28%
ssc00982:Drug metabolism	8	−0.39	−0.92	0.575	0.79	1	16	tags = 25%, list = 5%, signal = 26%
ssc00830:Retinol metabolism	3	−0.52	−0.92	0.608	0.782	1	38	tags = 67%, list = 11%, signal = 74%
ssc00531:Glycosaminoglycan degradation	3	−0.52	−0.91	0.604	0.786	1	135	tags = 67%, list = 39%, signal = 109%
ssc05014:Amyotrophic lateral sclerosis (ALS)	10	−0.36	−0.9	0.576	0.789	1	186	tags = 80%, list = 54%, signal = 169%
ssc04310:Wnt signaling pathway	15	−0.32	−0.89	0.615	0.789	1	64	tags = 27%, list = 19%, signal = 31%
ssc04062:Chemokine signaling pathway	18	−0.3	−0.88	0.644	0.807	1	134	tags = 61%, list = 39%, signal = 95%
ssc04150:mTOR signaling pathway	6	−0.4	−0.86	0.648	0.824	1	45	tags = 33%, list = 13%, signal = 38%

Table S4. *Cont.*

Pathway	Size	ES	NES	NOM *p*-val	FDR *q*-val	FWER *p*-val	Rank At Max	Leading Edge
ssc04110:Cell cycle	14	−0.31	−0.86	0.643	0.817	1	138	tags = 57%, list = 40%, signal = 92%
ssc00270:Cysteine and methionine metabolism	5	−0.42	−0.85	0.666	0.816	1	171	tags = 80%, list = 50%, signal = 157%
ssc05218:Melanoma	11	−0.32	−0.83	0.689	0.845	1	45	tags = 27%, list = 13%, signal = 30%
ssc04620:Toll-like receptor signaling pathway	17	−0.28	−0.83	0.701	0.835	1	65	tags = 29%, list = 19%, signal = 34%
ssc05332:Graft-versus-host disease	10	−0.33	−0.82	0.69	0.835	1	31	tags = 20%, list = 9%, signal = 21%
ssc04670:Leukocyte transendothelial migration	11	−0.32	−0.82	0.702	0.829	1	119	tags = 45%, list = 35%, signal = 67%
ssc00052:Galactose metabolism	3	−0.46	−0.82	0.726	0.821	1	159	tags = 67%, list = 46%, signal = 123%
ssc05219:Bladder cancer	8	−0.35	−0.81	0.718	0.823	1	35	tags = 25%, list = 10%, signal = 27%
ssc00400:Phenylalanine, tyrosine and tryptophan biosynthesis	2	−0.51	−0.8	0.776	0.828	1	171	tags = 100%, list = 50%, signal = 198%
ssc04120:Ubiquitin mediated proteolysis	9	−0.31	−0.79	0.729	0.838	1	158	tags = 56%, list = 46%, signal = 100%
ssc00590:Arachidonic acid metabolism	5	−0.38	−0.78	0.772	0.84	1	15	tags = 20%, list = 4%, signal = 21%
ssc04666:Fc gamma R-mediated phagocytosis	7	−0.33	−0.77	0.772	0.847	1	77	tags = 29%, list = 22%, signal = 36%
ssc00510:N-Glycan biosynthesis	6	−0.35	−0.76	0.781	0.85	1	228	tags = 100%, list = 66%, signal = 291%
ssc04930:Type II diabetes mellitus	6	−0.35	−0.74	0.801	0.863	1	45	tags = 33%, list = 13%, signal = 38%
ssc05016:HuntinTGon's disease	18	−0.24	−0.71	0.833	0.892	1	181	tags = 61%, list = 53%, signal = 122%
ssc04115:p53 signaling pathway	14	−0.25	−0.69	0.847	0.896	1	182	tags = 71%, list = 53%, signal = 146%
ssc00770:Pantothenate and CoA biosynthesis	2	−0.44	−0.69	0.91	0.895	1	95	tags = 50%, list = 28%, signal = 69%
ssc00630:Glyoxylate and dicarboxylate metabolism	3	−0.36	−0.64	0.924	0.927	1	221	tags = 100%, list = 64%, signal = 277%
ssc00030:Pentose phosphate pathway	2	−0.4	−0.62	0.957	0.935	1	53	tags = 50%, list = 15%, signal = 59%
ssc04622:RIG-I-like receptor signaling pathway	14	−0.22	−0.62	0.917	0.929	1	145	tags = 57%, list = 42%, signal = 95%
ssc00601:Glycosphingolipid biosynthesis	2	−0.31	−0.49	1	0.983	1	101	tags = 50%, list = 29%, signal = 70%

Table S5. Pathways enriched in phenotype CG.

Pathways	Size	ES	NES	NOM *p*-val	FDR *q*-val	FWER *p*-val	Rank at Max	Leading Edge
ssc03010:Ribosome	7	0.68	1.79	0.009	0.358	0.355	114	tags = 100%, list = 33%, signal = 147%
ssc05310:Asthma	7	0.66	1.73	0.027	0.284	0.521	68	tags = 86%, list = 20%, signal = 105%
ssc05330:Allograft rejection	11	0.54	1.7	0.025	0.221	0.575	80	tags = 82%, list = 23%, signal = 103%
ssc04940:Type I diabetes mellitus	10	0.52	1.6	0.036	0.307	0.784	80	tags = 80%, list = 23%, signal = 101%
ssc04260:Cardiac muscle contraction	6	0.63	1.6	0.039	0.25	0.791	53	tags = 50%, list = 15%, signal = 58%
ssc05320:Autoimmune thyroid disease	9	0.53	1.53	0.074	0.309	0.908	80	tags = 89%, list = 23%, signal = 113%
ssc04060:Cytokine-cytokine receptor interaction	12	0.43	1.45	0.084	0.42	0.978	61	tags = 58%, list = 18%, signal = 68%
ssc04672:Intestinal immune network for IgA production	9	0.48	1.41	0.108	0.451	0.994	68	tags = 78%, list = 20%, signal = 94%
ssc00230:Purine metabolism	4	0.66	1.39	0.094	0.434	0.995	28	tags = 50%, list = 8%, signal = 54%
ssc02010:ABC transporters	2	0.81	1.34	0.139	0.513	0.997	66	tags = 100%, list = 19%, signal = 123%
ssc05412:Arrhythmogenic right ventricular cardiomyopathy (ARVC)	2	0.77	1.25	0.21	0.692	1	26	tags = 50%, list = 8%, signal = 54%
ssc05322:Systemic lupus erythematosus	12	0.36	1.17	0.265	0.845	1	68	tags = 58%, list = 20%, signal = 70%
ssc04612:Antigen processing and presentation	17	0.31	1.16	0.243	0.791	1	85	tags = 65%, list = 25%, signal = 82%
ssc05414:Dilated cardiomyopathy	6	0.45	1.15	0.282	0.777	1	29	tags = 33%, list = 8%, signal = 36%
ssc04340:Hedgehog signaling pathway	4	0.52	1.14	0.278	0.737	1	31	tags = 50%, list = 9%, signal = 54%
ssc04512:ECM-receptor interaction	3	0.55	1.07	0.364	0.859	1	23	tags = 67%, list = 7%, signal = 71%
ssc00240:Pyrimidine metabolism	3	0.54	1.03	0.42	0.905	1	158	tags = 100%, list = 46%, signal = 183%
ssc04730:Long-term depression	5	0.44	1.03	0.392	0.855	1	0	tags = 20%, list = 0%, signal = 20%
ssc04514:Cell adhesion molecules (CAMs)	16	0.28	1.01	0.411	0.861	1	80	tags = 56%, list = 23%, signal = 70%
ssc00760:Nicotinate and nicotinamide metabolism	2	0.61	1	0.465	0.849	1	134	tags = 100%, list = 39%, signal = 163%
ssc04350:TGF-beta signaling pathway	14	0.28	0.96	0.465	0.901	1	67	tags = 36%, list = 19%, signal = 43%

Table S5. *Cont.*

Pathways	Size	ES	NES	NOM *p*-val	FDR *q*-val	FWER *p*-val	Rank at Max	Leading Edge
ssc05012:Parkinson's disease	12	0.28	0.94	0.528	0.904	1	251	tags = 100%, list = 73%, signal = 357%
ssc00562:Inositol phosphate metabolism	4	0.44	0.94	0.513	0.878	1	8	tags = 25%, list = 2%, signal = 25%
ssc00250:Alanine, aspartate and glutamate metabolism	5	0.39	0.93	0.541	0.864	1	16	tags = 40%, list = 5%, signal = 41%
ssc04130:SNARE interactions in vesicular transport	2	0.55	0.91	0.564	0.859	1	156	tags = 100%, list = 45%, signal = 182%
ssc04140:Regulation of autophagy	2	0.54	0.9	0.583	0.86	1	14	tags = 50%, list = 4%, signal = 52%
ssc00062:Fatty acid elongation in mitochondria	2	0.53	0.87	0.633	0.877	1	162	tags = 100%, list = 47%, signal = 188%
ssc05217:Basal cell carcinoma	6	0.34	0.87	0.605	0.846	1	31	tags = 33%, list = 9%, signal = 36%
ssc00983:Drug metabolism	3	0.45	0.87	0.639	0.829	1	4	tags = 33%, list = 1%, signal = 33%
ssc05416:Viral myocarditis	15	0.24	0.84	0.636	0.842	1	80	tags = 53%, list = 23%, signal = 66%
ssc05410:Hypertrophic cardiomyopathy (HCM)	8	0.3	0.82	0.72	0.847	1	26	tags = 25%, list = 8%, signal = 26%
ssc04520:Adherens junction	10	0.27	0.82	0.675	0.826	1	44	tags = 30%, list = 13%, signal = 33%
ssc04960:Aldosterone-regulated sodium reabsorption	3	0.42	0.8	0.722	0.833	1	13	tags = 33%, list = 4%, signal = 34%
ssc00480:Glutathione metabolism	8	0.28	0.8	0.731	0.812	1	18	tags = 25%, list = 5%, signal = 26%
ssc05340:Primary immunodeficiency	3	0.41	0.79	0.766	0.81	1	61	tags = 67%, list = 18%, signal = 80%
ssc03020:RNA polymerase	13	0.22	0.77	0.769	0.81	1	25	tags = 15%, list = 7%, signal = 16%
ssc04320:Dorso-ventral axis formation	2	0.38	0.6	0.973	0.97	1	213	tags = 100%, list = 62%, signal = 261%
ssc00051:Fructose and mannose metabolism	4	0.26	0.57	0.955	0.962	1	32	tags = 25%, list = 9%, signal = 27%

© 2013 by the authors; licensee MDPI, Basel, Switzerland. This article is an open access article distributed under the terms and conditions of the Creative Commons Attribution license (http://creativecommons.org/licenses/by/3.0/).
